# Acute small bowel obstruction secondary to a right-sided paraduodenal hernia: a case report and review of operative management

**DOI:** 10.1093/jscr/rjag752

**Published:** 2026-08-30

**Authors:** Rachelle Teoh, Rhys McClen, Dragos Iorgulescu

**Affiliations:** General Surgery Department, Shoalhaven District Memorial Hospital, Nowra 2541, NSW, Australia; General Surgery Department, Shoalhaven District Memorial Hospital, Nowra 2541, NSW, Australia; General Surgery Department, Shoalhaven District Memorial Hospital, Nowra 2541, NSW, Australia

**Keywords:** right sided paraduodenal hernia, bowel obstruction, surgical management

## Abstract

Paraduodenal hernias (PDH) are the most common type of internal hernia, accounting for ~53% of cases. Right-sided PDH are considerably less common than left-sided variants, comprising approximately one quarter of all PDH. Approximately 50% of PDH may progress to bowel obstruction during a patient’s lifetime, although PDH account for <1% of all small bowel obstructions. Pre-operative diagnosis is challenging due to the non-specific clinical presentation, with computed tomography imaging representing the diagnostic modality of choice. We present the case of an 18-year-old male with acute abdominal pain and vomiting secondary to a right-sided PDH causing high-grade small bowel obstruction. The patient underwent successful operative management with an uncomplicated post-operative recovery. This case emphasizes the importance of early recognition and timely surgical intervention in preventing bowel compromise and associated morbidity.

## Introduction

Paraduodenal hernias (PDH) arise secondary to a congenital defect involving either the fossa of Waldeyer (right-sided) or the fossa of Landzert (left-sided) [1]. Right-sided PDH occur due to failure of the pre-arterial midgut rotation around the superior mesenteric artery, whereas left-sided variants result from the herniation of small bowel between the inferior mesenteric vein and the posterior attachment of the mesocolon to the retroperitoneum [2]. Clinical diagnosis is difficult, as patients can present with a wide spectrum of symptoms ranging from chronic non-specific abdominal pain to acute small bowel obstruction [3, 4]. Consequently, a high index of suspicion is needed. Cross-sectional imaging plays an important role in differentiating PDH from other causes of bowel obstruction and assists with pre-operative diagnosis and planning. Furthermore, oral contrast may assist in distinguishing incarcerated bowel loops from solid intra-abdominal masses [3].

## Case report

**Figure 1:**
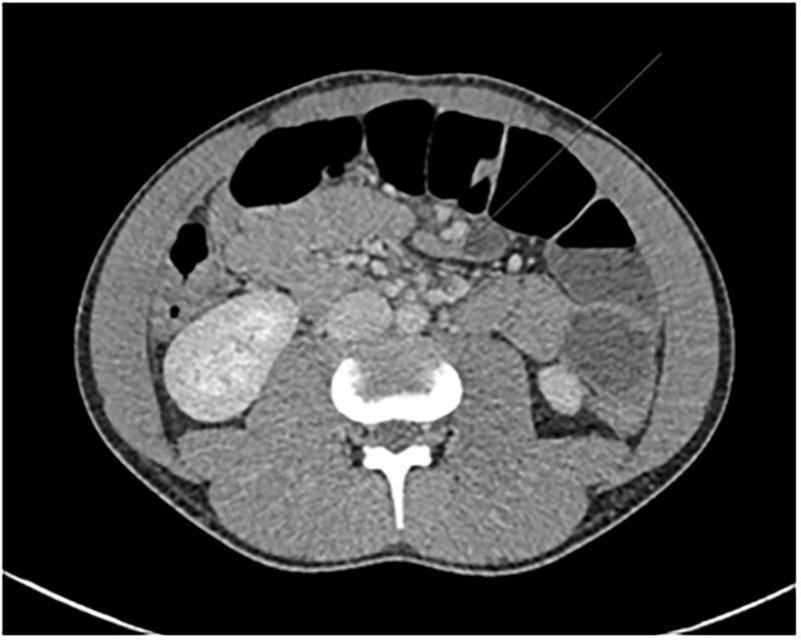
Axial view of right para-duodenal hernia transition point.

**Figure 2:**
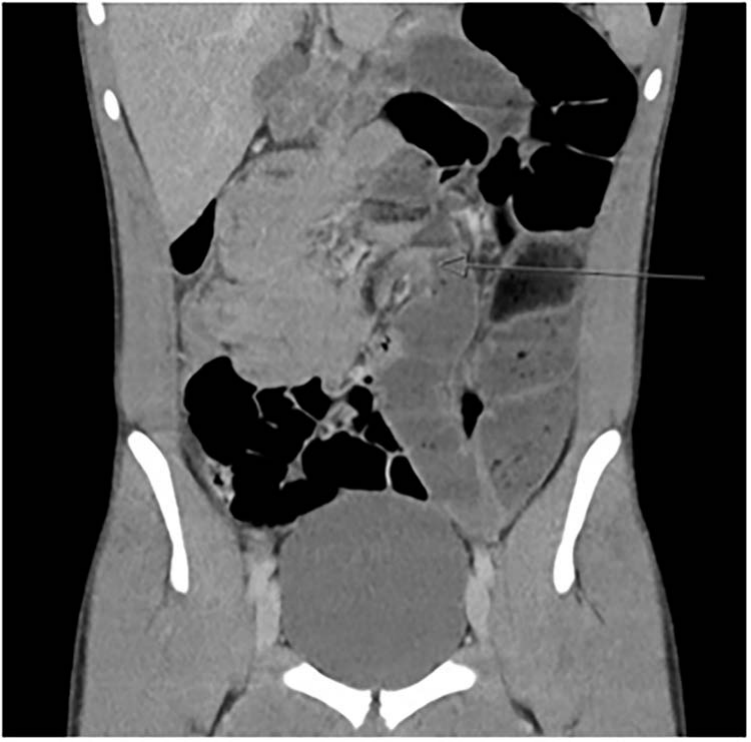
Coronal view of right para-duodenal hernia transition point.

An 18-year-old previously well male presented with sudden-onset severe generalized abdominal pain that subsequently localized to the epigastrium and lower abdomen, associated with profuse vomiting. He was otherwise previously well, with no prior abdominal surgery or significant medical history. Examination demonstrated maximal tenderness over the epigastrium without features of peritonism. He had an elevated white cell count of 21 ×10^9^/L and a lactate of 4 mmol/L on presentation. He underwent a computed tomography (CT) abdomen and pelvis with delayed venous phase intravenous contrast, which demonstrated a right-sided PDH causing high-grade small bowel obstruction (Figs 1 and 2). Pre-operatively, a nasogastric tube was also inserted as well as an indwelling catheter (IDC).

**Figure 3:**
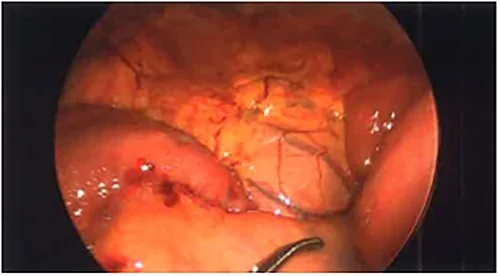
Right sided para-duodenal hernia herniating into fossa of Waldeyer.

**Figure 4:**
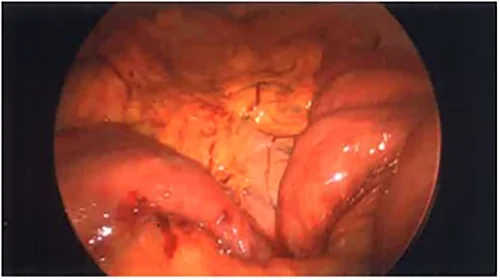
Right sided para-duodenal hernia herniating into fossa of Waldeyer.

**Figure 5:**
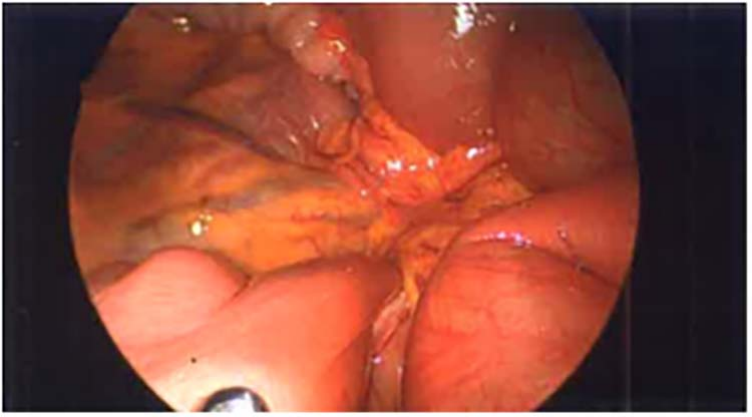
Right sided para-duodenal hernia herniating into fossa of Waldeyer.

The patient subsequently underwent a diagnostic laparoscopy during which he was found to have a right-sided PDH through a mesenteric defect in front of the superior mesenteric vein with a long closed-loop segment of small bowel herniating from left to right into the fossa of Waldeyer (Figs 3-5). The bowel was viable, and a small volume of serous free fluid was noted intra-operatively. The small bowel was examined from terminal ileum to the duodenojejunal flexure, and the hernia was able to be reduced. Conversion to upper midline laparotomy was required due to limited visualization of the hernia defect and challenging retraction of multiple dilated small bowel loops. The hernia defect was then closed with 4–0 PDS sutures to reduce the risk of recurrence. A prophylactic appendicectomy was also performed to avoid future diagnostic uncertainty. The patient recovered well with no complications and was discharged on post-operative day 5. His IDC was removed on post-operative day 1 and his NGT was removed on post-operative day 3.

## Discussion

Clinical presentation of PDH is highly variable, ranging from chronic intermittent abdominal pain to acute small bowel obstruction [1, 3]. As such, a high index of suspicion is required for diagnosis, particularly in young patients without previous abdominal surgery [3–5]. CT is the imaging modality of choice, as it facilitates both pre-operative diagnosis and operative planning [2, 3, 6]. In the present case, CT imaging demonstrated a right-sided PDH causing high-grade small bowel obstruction in the setting of acute obstructive symptoms.

There is currently no consensus regarding the optimal management of PDH; however, the literature generally supports operative intervention due to the risk of obstruction, strangulation, and bowel ischaemia [2, 5, 7]. Both laparoscopic and open approaches have been described [2, 7]. Several reports suggest that a laparoscopic approach may provide improved post-operative recovery while also allowing diagnostic confirmation, although conversion to laparotomy may be necessary in technically difficult cases [8]. A literature review by Oshita et al. further suggested that right-sided PDH more commonly present emergently and may be more difficult to reduce compared with left-sided variants, with some reports describing division of the inferior mesenteric vein to facilitate reduction [8].

In the present case, a laparoscopic approach was initially undertaken, with intra-operative findings confirming the CT diagnosis and successful laparoscopic reduction of the hernia contents. However, due to limited visualization of the hernia defect and difficulty retracting markedly dilated small bowel loops, conversion to an upper midline laparotomy was required to facilitate definitive repair. The patient’s acute presentation was also consistent with previously reported findings regarding right-sided PDH [8].

Management of the hernia orifice remains controversial and there is a relative paucity of literature regarding optimal operative technique. Some authors advocate widening the defect rather than primary closure due to the close relationship with the superior mesenteric vessels and concerns regarding tissue fragility [6, 8]. Conversely, others favour primary closure to reduce the risk of recurrence. Oshita et al. reported that six of eight cases reviewed for laparoscopic right-sided PDH repair elected to widen the hernia orifice laparoscopically rather than close the defect [6]. The same authors also cited an estimated lifetime obstruction risk of ~50% in untreated PDH, supporting surgical management [6].

Primary closure of the defect has nevertheless been successfully described using both laparoscopic and open techniques [1, 2, 4, 7–9]. Additional operative strategies described in the literature include excision of the hernia sac followed by defect closure [2]. In the present case, primary closure of the defect was performed to minimize the risk of recurrence, particularly given the patient’s young age.

A Chinese study proposed a classification system for right-sided PDH based on anatomical characteristics, including normal duodenal morphology, absence of the ligament of Treitz with passage of the efferent loop through the hernia defect, and the presence of Ladd’s bands extending from the caecum to the peritoneum or liver. The authors additionally proposed different operative approaches according to these anatomical subtypes [9]. While such classification systems may help guide future operative management, there remains no formalized guideline or consensus regarding the optimal surgical management of right-sided PDH [2].

## Conclusion

Right-sided PDH are a rare cause of small bowel obstruction and remain challenging to diagnose pre-operatively due to their non-specific presentation. CT imaging is essential for diagnosis and operative planning. Although no consensus exists regarding the optimal operative technique, operative management should be individualized according to intra-operative findings and surgeon experience.
